# 2-(2-Bromo­eth­yl)isoindoline-1,3-dione

**DOI:** 10.1107/S1600536811039808

**Published:** 2011-10-05

**Authors:** Xiao-Qiang Sun, Dong An, Ying Shao

**Affiliations:** a Key Laboratory of Fine Petrochemical Engineering, Changzhou University, Changzhou 213164, Jiangsu, People’s Republic of China

## Abstract

The asymmetric unit of the title compound, C_10_H_8_BrNO_2_, contains three crystallographically independent mol­ecules. Two of the N—C—C—Br side chains adopt *anti* conformations [torsion angles = −179.8 (5) and −179.4 (4)°] and the other is *gauche* [−66.5 (6)°]. The crystal structure features short Br⋯O [3.162 (5) Å] contacts, C—H⋯O hydrogen bonds and numerous π–π stacking inter­actions [centroid–centroid separations = 3.517 (4)–3.950 (4) Å].

## Related literature

For general background to and applications of the title compound, see: Sheng *et al.* (2007 ▶). For the preparation, see: Clouet & Juhl (1994 ▶).
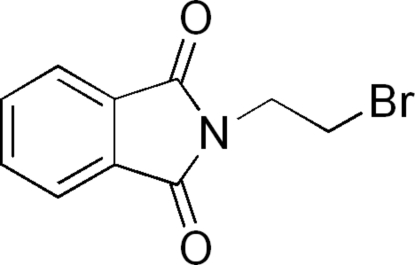


## Experimental

### 

#### Crystal data

C_10_H_8_BrNO_2_
*M* *_r_* = 254.08
Triclinic, 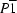

*a* = 8.575 (2) Å
*b* = 11.067 (3) Å
*c* = 16.333 (5) Å
α = 99.001 (6)°
β = 96.164 (5)°
γ = 102.259 (6)°
*V* = 1480.2 (7) Å^3^
*Z* = 6
Mo *K*α radiation
μ = 4.14 mm^−1^
*T* = 297 K
0.25 × 0.23 × 0.20 mm

#### Data collection

Bruker APEXII CCD diffractometer
Absorption correction: multi-scan (*SADABS*; Bruker, 2000 ▶) *T*_min_ = 0.370, *T*_max_ = 0.437
7716 measured reflections
5043 independent reflections
3241 reflections with *I* > 2σ(*I*)
*R*_int_ = 0.042

#### Refinement

*R*[*F* ^2^ > 2σ(*F* ^2^)] = 0.061
*wR*(*F* ^2^) = 0.177
*S* = 1.00
5043 reflections
379 parameters
H-atom parameters constrained
Δρ_max_ = 0.83 e Å^−3^
Δρ_min_ = −0.64 e Å^−3^

### 

Data collection: *APEX2* (Bruker, 2000 ▶); cell refinement: *SAINT* (Bruker, 2000 ▶); data reduction: *SAINT*; program(s) used to solve structure: *SHELXS97* (Sheldrick, 2008 ▶); program(s) used to refine structure: *SHELXL97* (Sheldrick, 2008 ▶); molecular graphics: *SHELXTL* (Sheldrick, 2008 ▶); software used to prepare material for publication: *SHELXTL*.

## Supplementary Material

Crystal structure: contains datablock(s) I, global. DOI: 10.1107/S1600536811039808/hb6417sup1.cif

Structure factors: contains datablock(s) I. DOI: 10.1107/S1600536811039808/hb6417Isup2.hkl

Supplementary material file. DOI: 10.1107/S1600536811039808/hb6417Isup3.cdx

Supplementary material file. DOI: 10.1107/S1600536811039808/hb6417Isup4.cml

Additional supplementary materials:  crystallographic information; 3D view; checkCIF report

## Figures and Tables

**Table 1:** Hydrogen-bond geometry (Å, °)

*D*—H⋯*A*	*D*—H	H⋯*A*	*D*⋯*A*	*D*—H⋯*A*
C2—H2⋯O6^i^	0.93	2.59	3.301 (9)	133
C10—H10*A*⋯O3^ii^	0.97	2.48	3.409 (8)	161
C10—H10*B*⋯O5^iii^	0.97	2.60	3.533 (9)	163
C13—H13⋯O4^iv^	0.93	2.52	3.448 (8)	175
C14—H14⋯O1	0.93	2.59	3.495 (10)	165

Symmetry codes: (i) 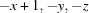; (ii) 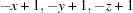; (iii) 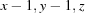; (iv) 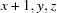.

**Fig. 1.**
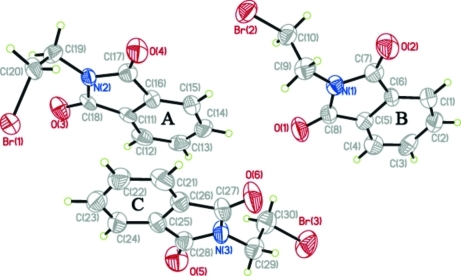
View of the title compound, showing 50% probability ellipsoids.

**Fig. 2.**
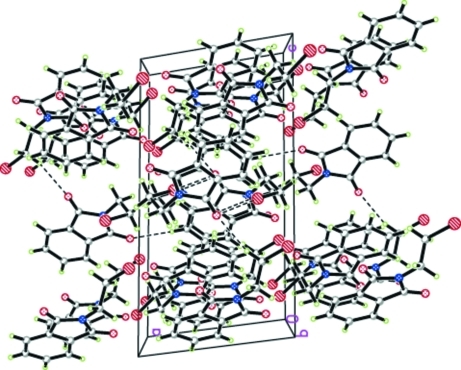
Perspective view of the packing of the title compound along b direction. Labels of atoms have been omitted for clarity.

## supplementary crystallographic information

### Comment

The title compound, C_10_H_8_BrNO_2_, which is an important aminoethylation reagent in the classic Gabriel Synthesis (Sheng *et al.*, 2007), was prepared by nucleophilic substitution of 1,2-dibromoethane with potassium phthalimide in DMF at room temperature (Clouet *et al.*, 1994). The title compound crystallizes in space group *P*1 with three crystallographically independent molecules in the asymmetric unit, designated *A*, *B* and *C* (Fig. 1). In the crystal, the phthalimide rings are almost coplanar [r.m.s. deviations = 0.0681 (*A*), 0.0125 (*B*), 0.0113 (*C*) Å, respectively]. The molecular geometries of all molecules are essentiall similar, except for the BrCH_2_CH_2_ groups adopting slightly different zigzag conformations.

An interesting feature of the crystal structure is the short non-hydrogen Br···O interactions (table 1), together with intra- and inter-molecular C—H···O hydrogen bonding interactions (table 2), which links the molecules into an extended three-dimensional network, as shown in Fig. 2. The crystal structure is further stabilized by intermolecular π–π stacking interactions invoving the benzene and maleinimide rings [centroid-centroid distances = 3.517 (4) - 3.950 (4) Å].

### Experimental

The title compound was prepared according to literature procedures (Clouet *et al.*, 1994). Yield: 86%.  Colourless blocks were obtained by slow evaporation of EtOH.

### Refinement

All the H atoms were placed in geometrically idealized positions and constrained to ride on their parent atoms, with C—H distances of 0.93–0.97 Å, and with *U*_iso_(H) = 1.2*U*_eq_(C).

### Crystal data

**Table:**

C_10_H_8_BrNO_2_	*Z* = 6
*M**_r_* = 254.08	*F*(000) = 756
Triclinic, *P*1	*D*_x_ = 1.710 Mg m^−^^3^
Hall symbol: -P 1	Melting point: 356.0(3) K
*a* = 8.575 (2) Å	Mo *K*α radiation, λ = 0.71073 Å
*b* = 11.067 (3) Å	Cell parameters from 3260 reflections
*c* = 16.333 (5) Å	θ = 2.5–27.1°
α = 99.001 (6)°	µ = 4.14 mm^−^^1^
β = 96.164 (5)°	*T* = 297 K
γ = 102.259 (6)°	Block, colorless
*V* = 1480.2 (7) Å^3^	0.25 × 0.23 × 0.20 mm

### Data collection

**Table:**

Bruker APEXII CCD diffractometer	5043 independent reflections
Radiation source: fine-focus sealed tube	3241 reflections with *I* > 2σ(*I*)
graphite	*R*_int_ = 0.042
φ and ω scans	θ_max_ = 25.0°, θ_min_ = 1.9°
Absorption correction: multi-scan (*SADABS*; Bruker, 2000)	*h* = −10→10
*T*_min_ = 0.370, *T*_max_ = 0.437	*k* = −7→13
7716 measured reflections	*l* = −19→19

### Refinement

**Table:**

Refinement on *F*^2^	Primary atom site location: structure-invariant direct methods
Least-squares matrix: full	Secondary atom site location: difference Fourier map
*R*[*F*^2^ > 2σ(*F*^2^)] = 0.061	Hydrogen site location: inferred from neighbouring sites
*wR*(*F*^2^) = 0.177	H-atom parameters constrained
*S* = 1.00	*w* = 1/[σ^2^(*F*_o_^2^) + (0.1033*P*)^2^] where *P* = (*F*_o_^2^ + 2*F*_c_^2^)/3
5043 reflections	(Δ/σ)_max_ < 0.001
379 parameters	Δρ_max_ = 0.83 e Å^−^^3^
0 restraints	Δρ_min_ = −0.64 e Å^−^^3^

### Special details

**Table:**

Geometry. All e.s.d.'s (except the e.s.d. in the dihedral angle between two l.s. planes) are estimated using the full covariance matrix. The cell e.s.d.'s are taken into account individually in the estimation of e.s.d.'s in distances, angles and torsion angles; correlations between e.s.d.'s in cell parameters are only used when they are defined by crystal symmetry. An approximate (isotropic) treatment of cell e.s.d.'s is used for estimating e.s.d.'s involving l.s. planes.
Refinement. Refinement of *F*^2^ against ALL reflections. The weighted *R*-factor *wR* and goodness of fit *S* are based on *F*^2^, conventional *R*-factors *R* are based on *F*, with *F* set to zero for negative *F*^2^. The threshold expression of *F*^2^ > σ(*F*^2^) is used only for calculating *R*-factors(gt) *etc*. and is not relevant to the choice of reflections for refinement. *R*-factors based on *F*^2^ are statistically about twice as large as those based on *F*, and *R*- factors based on ALL data will be even larger.

### Fractional atomic coordinates and isotropic or equivalent isotropic displacement parameters (Å^2^)

**Table:**

	*x*	*y*	*z*	*U*_iso_*/*U*_eq_	
Br2	0.05219 (9)	0.17632 (7)	0.29910 (5)	0.0612 (3)	
Br1	0.22870 (8)	0.96923 (6)	0.46532 (5)	0.0495 (2)	
Br3	1.05699 (13)	0.37156 (10)	0.10602 (7)	0.0923 (4)	
C1	0.4862 (9)	−0.2168 (6)	0.0232 (4)	0.0491 (17)	
H1	0.3998	−0.2820	−0.0034	0.059*	
C2	0.6406 (9)	−0.2208 (7)	0.0145 (4)	0.0538 (19)	
H2	0.6596	−0.2922	−0.0173	0.065*	
C3	0.7710 (9)	−0.1236 (8)	0.0510 (5)	0.059 (2)	
H3	0.8748	−0.1300	0.0428	0.070*	
C4	0.7475 (8)	−0.0169 (7)	0.0997 (5)	0.0528 (18)	
H4	0.8346	0.0492	0.1242	0.063*	
C5	0.5920 (7)	−0.0105 (6)	0.1114 (4)	0.0393 (15)	
C6	0.4630 (8)	−0.1100 (5)	0.0740 (4)	0.0382 (15)	
C7	0.3144 (8)	−0.0779 (6)	0.0967 (4)	0.0441 (16)	
C8	0.5291 (8)	0.0885 (6)	0.1586 (4)	0.0443 (16)	
C9	0.2475 (8)	0.1140 (7)	0.1804 (5)	0.0511 (18)	
H9A	0.2939	0.2036	0.1878	0.061*	
H9B	0.1499	0.0940	0.1400	0.061*	
C10	0.2073 (8)	0.0820 (7)	0.2603 (4)	0.0503 (17)	
H10A	0.3041	0.1032	0.3013	0.060*	
H10B	0.1612	−0.0075	0.2533	0.060*	
C11	0.5109 (7)	0.6382 (5)	0.4534 (4)	0.0365 (14)	
C12	0.6682 (8)	0.6298 (6)	0.4518 (5)	0.0507 (18)	
H12	0.7518	0.6849	0.4893	0.061*	
C13	0.6980 (8)	0.5376 (7)	0.3930 (5)	0.054 (2)	
H13	0.8037	0.5308	0.3906	0.065*	
C14	0.5758 (9)	0.4553 (7)	0.3379 (5)	0.0532 (19)	
H14	0.6008	0.3943	0.2989	0.064*	
C15	0.4148 (9)	0.4604 (6)	0.3386 (5)	0.0514 (18)	
H15	0.3311	0.4043	0.3016	0.062*	
C16	0.3882 (7)	0.5545 (6)	0.3979 (4)	0.0371 (14)	
C17	0.2320 (7)	0.5819 (6)	0.4174 (4)	0.0389 (15)	
C18	0.4407 (7)	0.7225 (5)	0.5093 (4)	0.0373 (15)	
C19	0.1552 (8)	0.7387 (6)	0.5261 (4)	0.0468 (17)	
H19A	0.2082	0.7887	0.5799	0.056*	
H19B	0.0719	0.6707	0.5364	0.056*	
C20	0.0780 (7)	0.8198 (5)	0.4764 (5)	0.0446 (17)	
H20A	−0.0089	0.8432	0.5038	0.054*	
H20B	0.0317	0.7716	0.4211	0.054*	
C21	0.3965 (9)	0.6962 (7)	0.2211 (5)	0.058 (2)	
H21	0.3061	0.6374	0.1915	0.069*	
C22	0.3827 (9)	0.8007 (7)	0.2763 (5)	0.057 (2)	
H22	0.2808	0.8113	0.2852	0.069*	
C23	0.5206 (9)	0.8916 (7)	0.3192 (5)	0.0545 (19)	
H23	0.5093	0.9624	0.3550	0.065*	
C24	0.6708 (9)	0.8747 (7)	0.3077 (4)	0.0510 (17)	
H24	0.7627	0.9332	0.3360	0.061*	
C25	0.6838 (8)	0.7710 (6)	0.2544 (4)	0.0412 (15)	
C26	0.5487 (8)	0.6835 (6)	0.2123 (4)	0.0433 (16)	
C27	0.6050 (8)	0.5849 (6)	0.1569 (5)	0.0495 (17)	
C28	0.8293 (8)	0.7294 (6)	0.2302 (4)	0.0450 (16)	
C29	0.8764 (10)	0.5529 (7)	0.1255 (5)	0.061 (2)	
H29A	0.9743	0.6128	0.1211	0.074*	
H29B	0.8219	0.5150	0.0693	0.074*	
C30	0.9173 (10)	0.4537 (7)	0.1703 (5)	0.064 (2)	
H30A	0.8199	0.3931	0.1744	0.077*	
H30B	0.9723	0.4911	0.2264	0.077*	
N1	0.3639 (6)	0.0434 (5)	0.1479 (3)	0.0419 (13)	
N2	0.2731 (6)	0.6854 (4)	0.4833 (3)	0.0363 (12)	
N3	0.7699 (7)	0.6185 (5)	0.1727 (4)	0.0514 (15)	
O1	0.6004 (6)	0.1893 (5)	0.1976 (4)	0.0694 (16)	
O2	0.1764 (6)	−0.1350 (5)	0.0784 (4)	0.0734 (17)	
O3	0.5045 (5)	0.8060 (4)	0.5681 (3)	0.0544 (13)	
O4	0.0969 (5)	0.5313 (5)	0.3867 (3)	0.0596 (14)	
O5	0.9687 (6)	0.7775 (5)	0.2544 (4)	0.0713 (16)	
O6	0.5281 (7)	0.4928 (5)	0.1101 (4)	0.0819 (19)	

### Atomic displacement parameters (Å^2^)

**Table:**

	*U*^11^	*U*^22^	*U*^33^	*U*^12^	*U*^13^	*U*^23^
Br2	0.0557 (5)	0.0705 (5)	0.0602 (5)	0.0213 (4)	0.0216 (4)	0.0028 (4)
Br1	0.0543 (4)	0.0379 (4)	0.0570 (5)	0.0104 (3)	0.0134 (3)	0.0080 (3)
Br3	0.1184 (8)	0.0883 (7)	0.0948 (7)	0.0682 (6)	0.0399 (6)	0.0136 (6)
C1	0.051 (4)	0.044 (4)	0.047 (4)	0.009 (3)	0.002 (3)	0.001 (3)
C2	0.061 (5)	0.053 (4)	0.047 (4)	0.017 (4)	0.011 (4)	0.002 (3)
C3	0.054 (5)	0.084 (6)	0.047 (4)	0.034 (4)	0.017 (4)	0.007 (4)
C4	0.036 (4)	0.064 (5)	0.054 (5)	0.010 (3)	0.002 (3)	0.004 (4)
C5	0.032 (3)	0.047 (4)	0.038 (4)	0.011 (3)	0.002 (3)	0.007 (3)
C6	0.044 (4)	0.032 (3)	0.036 (4)	0.008 (3)	0.003 (3)	0.000 (3)
C7	0.030 (4)	0.059 (4)	0.037 (4)	0.005 (3)	−0.003 (3)	0.006 (3)
C8	0.045 (4)	0.040 (4)	0.046 (4)	0.009 (3)	0.007 (3)	0.002 (3)
C9	0.046 (4)	0.053 (4)	0.061 (5)	0.025 (3)	0.010 (4)	0.012 (4)
C10	0.048 (4)	0.054 (4)	0.050 (4)	0.017 (3)	0.010 (3)	0.003 (3)
C11	0.028 (3)	0.028 (3)	0.052 (4)	0.001 (2)	0.002 (3)	0.012 (3)
C12	0.031 (4)	0.043 (4)	0.078 (5)	0.002 (3)	0.005 (4)	0.020 (4)
C13	0.043 (4)	0.054 (4)	0.082 (6)	0.023 (4)	0.031 (4)	0.032 (4)
C14	0.061 (5)	0.046 (4)	0.064 (5)	0.022 (4)	0.030 (4)	0.014 (4)
C15	0.061 (5)	0.035 (4)	0.061 (5)	0.013 (3)	0.020 (4)	0.006 (3)
C16	0.038 (3)	0.033 (3)	0.040 (4)	0.008 (3)	0.004 (3)	0.006 (3)
C17	0.028 (3)	0.041 (4)	0.044 (4)	0.005 (3)	0.000 (3)	0.005 (3)
C18	0.041 (4)	0.021 (3)	0.048 (4)	0.002 (3)	0.003 (3)	0.010 (3)
C19	0.038 (4)	0.051 (4)	0.052 (4)	0.010 (3)	0.016 (3)	0.005 (3)
C20	0.027 (3)	0.032 (3)	0.073 (5)	0.011 (3)	0.005 (3)	−0.001 (3)
C21	0.048 (4)	0.045 (4)	0.078 (6)	0.005 (3)	0.008 (4)	0.016 (4)
C22	0.058 (5)	0.058 (5)	0.069 (5)	0.027 (4)	0.026 (4)	0.021 (4)
C23	0.067 (5)	0.054 (4)	0.051 (4)	0.024 (4)	0.019 (4)	0.015 (4)
C24	0.057 (4)	0.050 (4)	0.045 (4)	0.013 (3)	0.009 (4)	0.004 (3)
C25	0.039 (4)	0.034 (3)	0.049 (4)	0.011 (3)	−0.003 (3)	0.006 (3)
C26	0.043 (4)	0.041 (4)	0.048 (4)	0.014 (3)	0.005 (3)	0.008 (3)
C27	0.048 (4)	0.040 (4)	0.051 (4)	0.007 (3)	−0.004 (3)	−0.007 (3)
C28	0.042 (4)	0.044 (4)	0.048 (4)	0.010 (3)	0.006 (3)	0.004 (3)
C29	0.070 (5)	0.059 (5)	0.060 (5)	0.023 (4)	0.020 (4)	0.006 (4)
C30	0.066 (5)	0.069 (5)	0.061 (5)	0.027 (4)	0.012 (4)	0.007 (4)
N1	0.040 (3)	0.039 (3)	0.050 (3)	0.018 (2)	0.008 (3)	0.004 (2)
N2	0.031 (3)	0.026 (2)	0.050 (3)	0.007 (2)	0.009 (2)	0.000 (2)
N3	0.050 (4)	0.041 (3)	0.058 (4)	0.012 (3)	0.009 (3)	−0.009 (3)
O1	0.060 (3)	0.050 (3)	0.080 (4)	0.001 (3)	0.005 (3)	−0.020 (3)
O2	0.045 (3)	0.078 (4)	0.083 (4)	0.006 (3)	−0.001 (3)	−0.010 (3)
O3	0.042 (3)	0.039 (3)	0.068 (3)	0.000 (2)	−0.016 (3)	−0.003 (2)
O4	0.027 (2)	0.059 (3)	0.075 (4)	0.000 (2)	−0.006 (2)	−0.017 (3)
O5	0.038 (3)	0.072 (4)	0.091 (4)	0.003 (3)	−0.001 (3)	−0.003 (3)
O6	0.074 (4)	0.057 (3)	0.094 (5)	0.008 (3)	−0.002 (3)	−0.028 (3)

### Geometric parameters (Å, °)

**Table:**

Br2—C10	1.955 (6)	C15—H15	0.9300
Br1—C20	1.917 (6)	C16—C17	1.490 (8)
Br3—C30	1.949 (7)	C17—O4	1.191 (7)
C1—C2	1.356 (10)	C17—N2	1.399 (8)
C1—C6	1.397 (9)	C18—O3	1.211 (7)
C1—H1	0.9300	C18—N2	1.407 (8)
C2—C3	1.381 (10)	C19—N2	1.460 (7)
C2—H2	0.9300	C19—C20	1.506 (9)
C3—C4	1.382 (10)	C19—H19A	0.9700
C3—H3	0.9300	C19—H19B	0.9700
C4—C5	1.382 (9)	C20—H20A	0.9700
C4—H4	0.9300	C20—H20B	0.9700
C5—C6	1.392 (9)	C21—C26	1.363 (10)
C5—C8	1.470 (9)	C21—C22	1.385 (10)
C6—C7	1.462 (9)	C21—H21	0.9300
C7—O2	1.199 (8)	C22—C23	1.413 (11)
C7—N1	1.418 (8)	C22—H22	0.9300
C8—O1	1.196 (8)	C23—C24	1.369 (10)
C8—N1	1.380 (8)	C23—H23	0.9300
C9—C10	1.463 (10)	C24—C25	1.360 (9)
C9—N1	1.485 (8)	C24—H24	0.9300
C9—H9A	0.9700	C25—C26	1.377 (9)
C9—H9B	0.9700	C25—C28	1.489 (9)
C10—H10A	0.9700	C26—C27	1.500 (9)
C10—H10B	0.9700	C27—O6	1.191 (8)
C11—C12	1.374 (9)	C27—N3	1.369 (9)
C11—C16	1.381 (9)	C28—O5	1.196 (8)
C11—C18	1.468 (9)	C28—N3	1.385 (8)
C12—C13	1.374 (10)	C29—N3	1.488 (9)
C12—H12	0.9300	C29—C30	1.490 (10)
C13—C14	1.368 (10)	C29—H29A	0.9700
C13—H13	0.9300	C29—H29B	0.9700
C14—C15	1.395 (10)	C30—H30A	0.9700
C14—H14	0.9300	C30—H30B	0.9700
C15—C16	1.381 (9)		
			
Cg1···Cg4^i^	3.517 (4)	Cg2···Cg6	3.950 (4)
Cg1···Cg6^ii^	3.629 (4)	Cg3···Cg4^iv^	3.603 (4)
Cg2···Cg5^iii^	3.558 (4)	Cg3···Cg5	3.843 (4)
			
C2—C1—C6	116.9 (7)	N2—C18—C11	105.7 (5)
C2—C1—H1	121.6	N2—C19—C20	112.6 (5)
C6—C1—H1	121.6	N2—C19—H19A	109.1
C1—C2—C3	122.9 (7)	C20—C19—H19A	109.1
C1—C2—H2	118.6	N2—C19—H19B	109.1
C3—C2—H2	118.6	C20—C19—H19B	109.1
C2—C3—C4	120.2 (7)	H19A—C19—H19B	107.8
C2—C3—H3	119.9	C19—C20—Br1	112.4 (4)
C4—C3—H3	119.9	C19—C20—H20A	109.1
C3—C4—C5	118.6 (7)	Br1—C20—H20A	109.1
C3—C4—H4	120.7	C19—C20—H20B	109.1
C5—C4—H4	120.7	Br1—C20—H20B	109.1
C4—C5—C6	120.0 (6)	H20A—C20—H20B	107.9
C4—C5—C8	131.4 (6)	C26—C21—C22	116.9 (7)
C6—C5—C8	108.7 (5)	C26—C21—H21	121.6
C5—C6—C1	121.5 (6)	C22—C21—H21	121.6
C5—C6—C7	108.2 (5)	C21—C22—C23	121.3 (7)
C1—C6—C7	130.3 (6)	C21—C22—H22	119.4
O2—C7—N1	123.6 (6)	C23—C22—H22	119.4
O2—C7—C6	131.0 (6)	C24—C23—C22	119.5 (7)
N1—C7—C6	105.4 (5)	C24—C23—H23	120.3
O1—C8—N1	124.9 (6)	C22—C23—H23	120.3
O1—C8—C5	129.2 (6)	C25—C24—C23	119.0 (7)
N1—C8—C5	105.8 (5)	C25—C24—H24	120.5
C10—C9—N1	110.8 (6)	C23—C24—H24	120.5
C10—C9—H9A	109.5	C24—C25—C26	121.1 (6)
N1—C9—H9A	109.5	C24—C25—C28	130.4 (6)
C10—C9—H9B	109.5	C26—C25—C28	108.5 (5)
N1—C9—H9B	109.5	C21—C26—C25	122.2 (6)
H9A—C9—H9B	108.1	C21—C26—C27	130.2 (7)
C9—C10—Br2	108.9 (5)	C25—C26—C27	107.5 (6)
C9—C10—H10A	109.9	O6—C27—N3	124.8 (7)
Br2—C10—H10A	109.9	O6—C27—C26	129.5 (7)
C9—C10—H10B	109.9	N3—C27—C26	105.6 (5)
Br2—C10—H10B	109.9	O5—C28—N3	125.8 (6)
H10A—C10—H10B	108.3	O5—C28—C25	129.1 (6)
C12—C11—C16	120.2 (6)	N3—C28—C25	105.1 (6)
C12—C11—C18	130.8 (6)	N3—C29—C30	109.2 (6)
C16—C11—C18	109.0 (5)	N3—C29—H29A	109.8
C13—C12—C11	117.9 (7)	C30—C29—H29A	109.8
C13—C12—H12	121.0	N3—C29—H29B	109.8
C11—C12—H12	121.0	C30—C29—H29B	109.8
C14—C13—C12	121.6 (6)	H29A—C29—H29B	108.3
C14—C13—H13	119.2	C29—C30—Br3	107.4 (5)
C12—C13—H13	119.2	C29—C30—H30A	110.2
C13—C14—C15	121.9 (7)	Br3—C30—H30A	110.2
C13—C14—H14	119.0	C29—C30—H30B	110.2
C15—C14—H14	119.0	Br3—C30—H30B	110.2
C16—C15—C14	115.4 (7)	H30A—C30—H30B	108.5
C16—C15—H15	122.3	C8—N1—C7	111.9 (5)
C14—C15—H15	122.3	C8—N1—C9	125.3 (5)
C15—C16—C11	123.0 (6)	C7—N1—C9	122.6 (5)
C15—C16—C17	128.8 (6)	C17—N2—C18	111.7 (5)
C11—C16—C17	108.1 (5)	C17—N2—C19	123.9 (5)
O4—C17—N2	123.9 (6)	C18—N2—C19	124.1 (5)
O4—C17—C16	130.7 (6)	C27—N3—C28	113.3 (6)
N2—C17—C16	105.5 (5)	C27—N3—C29	123.9 (6)
O3—C18—N2	123.8 (6)	C28—N3—C29	122.4 (6)
O3—C18—C11	130.5 (6)		
			
C6—C1—C2—C3	−2.2 (11)	C22—C21—C26—C25	−1.4 (11)
C1—C2—C3—C4	0.9 (12)	C22—C21—C26—C27	−178.0 (7)
C2—C3—C4—C5	0.5 (11)	C24—C25—C26—C21	0.6 (11)
C3—C4—C5—C6	−0.4 (11)	C28—C25—C26—C21	−179.5 (7)
C3—C4—C5—C8	−179.7 (7)	C24—C25—C26—C27	177.9 (6)
C4—C5—C6—C1	−1.0 (10)	C28—C25—C26—C27	−2.2 (8)
C8—C5—C6—C1	178.4 (6)	C21—C26—C27—O6	−2.9 (14)
C4—C5—C6—C7	−179.9 (6)	C25—C26—C27—O6	−179.9 (8)
C8—C5—C6—C7	−0.5 (7)	C21—C26—C27—N3	179.4 (8)
C2—C1—C6—C5	2.2 (10)	C25—C26—C27—N3	2.4 (8)
C2—C1—C6—C7	−179.0 (7)	C24—C25—C28—O5	2.0 (13)
C5—C6—C7—O2	179.6 (8)	C26—C25—C28—O5	−177.9 (8)
C1—C6—C7—O2	0.8 (13)	C24—C25—C28—N3	−178.9 (7)
C5—C6—C7—N1	0.3 (7)	C26—C25—C28—N3	1.2 (8)
C1—C6—C7—N1	−178.5 (7)	N3—C29—C30—Br3	−179.8 (5)
C4—C5—C8—O1	1.1 (14)	O1—C8—N1—C7	178.5 (7)
C6—C5—C8—O1	−178.2 (7)	C5—C8—N1—C7	−0.3 (8)
C4—C5—C8—N1	179.8 (7)	O1—C8—N1—C9	2.3 (12)
C6—C5—C8—N1	0.5 (8)	C5—C8—N1—C9	−176.5 (6)
N1—C9—C10—Br2	−179.4 (4)	O2—C7—N1—C8	−179.3 (7)
C16—C11—C12—C13	0.8 (10)	C6—C7—N1—C8	0.0 (7)
C18—C11—C12—C13	177.9 (6)	O2—C7—N1—C9	−3.1 (11)
C11—C12—C13—C14	−0.5 (11)	C6—C7—N1—C9	176.3 (6)
C12—C13—C14—C15	−0.2 (11)	C10—C9—N1—C8	−96.1 (8)
C13—C14—C15—C16	0.6 (11)	C10—C9—N1—C7	88.1 (8)
C14—C15—C16—C11	−0.3 (10)	O4—C17—N2—C18	177.6 (6)
C14—C15—C16—C17	−177.0 (6)	C16—C17—N2—C18	−2.4 (7)
C12—C11—C16—C15	−0.4 (10)	O4—C17—N2—C19	4.2 (10)
C18—C11—C16—C15	−178.1 (6)	C16—C17—N2—C19	−175.8 (6)
C12—C11—C16—C17	176.9 (6)	O3—C18—N2—C17	−176.3 (6)
C18—C11—C16—C17	−0.7 (7)	C11—C18—N2—C17	2.0 (7)
C15—C16—C17—O4	−1.0 (12)	O3—C18—N2—C19	−2.9 (9)
C11—C16—C17—O4	−178.1 (7)	C11—C18—N2—C19	175.3 (6)
C15—C16—C17—N2	179.0 (6)	C20—C19—N2—C17	−78.0 (8)
C11—C16—C17—N2	1.9 (7)	C20—C19—N2—C18	109.4 (7)
C12—C11—C18—O3	0.1 (12)	O6—C27—N3—C28	−179.5 (8)
C16—C11—C18—O3	177.4 (6)	C26—C27—N3—C28	−1.7 (8)
C12—C11—C18—N2	−178.0 (7)	O6—C27—N3—C29	8.0 (13)
C16—C11—C18—N2	−0.7 (7)	C26—C27—N3—C29	−174.2 (6)
N2—C19—C20—Br1	−66.5 (6)	O5—C28—N3—C27	179.6 (7)
C26—C21—C22—C23	1.9 (11)	C25—C28—N3—C27	0.4 (8)
C21—C22—C23—C24	−1.6 (11)	O5—C28—N3—C29	−7.8 (12)
C22—C23—C24—C25	0.7 (11)	C25—C28—N3—C29	173.1 (6)
C23—C24—C25—C26	−0.2 (11)	C30—C29—N3—C27	−93.1 (9)
C23—C24—C25—C28	179.9 (7)	C30—C29—N3—C28	95.1 (8)

Symmetry codes: (i) −*x*+1, −*y*, −*z*; (ii) *x*, *y*−1, *z*; (iii) −*x*+1, −*y*+1, −*z*+1;  (iv) *x*, *y*+1, *z*.

### Hydrogen-bond geometry (Å, °)

**Table:**

*D*—H···*A*	*D*—H	H···*A*	*D*···*A*	*D*—H···*A*
C2—H2···O6^i^	0.93	2.59	3.301 (9)	133
C10—H10A···O3^iii^	0.97	2.48	3.409 (8)	161
C10—H10B···O5^v^	0.97	2.60	3.533 (9)	163
C13—H13···O4^vi^	0.93	2.52	3.448 (8)	175
C14—H14···O1	0.93	2.59	3.495 (10)	165

Symmetry codes: (i) −*x*+1, −*y*, −*z*; (iii) −*x*+1, −*y*+1, −*z*+1; (v) *x*−1, *y*−1, *z*; (vi) *x*+1, *y*, *z*.
